# Temporal Step Supervision for Robust Step Counting from Wearable Accelerometers

**DOI:** 10.3390/s26196235

**Published:** 2026-09-30

**Authors:** Yan Zhang, Paula Lago, Quoc Dinh Nguyen

**Affiliations:** 1 Centre de Recherche du CHUM, Montréal, QC H2X 0A9, Canada; yan.zhang.5@umontreal.ca; 2 Département d’Informatique et de Recherche Opérationnelle, Université de Montréal, Montréal, QC H3C 3J7, Canada; 3 Mila–Quebec AI Institute, Montréal, QC H2S 3H1, Canada; 4 Department of Electrical and Computer Engineering, Concordia University, Montréal, QC H3G 1M8, Canada; paula.lago@concordia.ca; 5 Département de Médecine, Faculté de Médecine, Université de Montréal, Montréal, QC H3C 3J7, Canada

**Keywords:** step counting, wearable accelerometers, temporal supervision, gait analysis, deep learning

## Abstract

Step-counting remains difficult in irregular walking, where step timing and signal patterns are less consistent than in controlled walking. We propose a step-counting model that uses step timing annotations as temporal supervision during training. Each step timestamp is expanded into a short temporal label interval, and a U-Net is used to localize step events because its dense prediction structure is well suited to temporal localization. The model is trained to estimate both where steps occur in the window and how many steps the window contains. The final count is obtained by summing the predicted values over time and dividing by the label width. On the Clemson wrist-worn dataset, the model achieves MAPE values of 4.61%, 9.90%, and 15.78% under regular, semi-regular, and irregular walking, respectively. Compared with classical step-detection algorithms and reproduced CNN, WaveNet, and LSTM baselines, the proposed model shows substantially lower error under irregular walking. On OxWalk, the model achieves 13.06% MAPE using only OxWalk training data, reaching performance comparable to fine-tuned Stepcount SSL, which relies on large-scale self-supervised pretraining. These results suggest that temporal step supervision improves robustness under irregular walking and can reduce dependence on large-scale external pretraining.

## 1. Introduction

Physical activity is strongly associated with health outcomes and is a central target in public health and rehabilitation monitoring [1,2]. Step count provides an intuitive measure of ambulatory physical activity because it is directly linked to the basic unit of human gait and is easier to interpret than aggregate acceleration or activity summaries derived from intensity thresholds [3]. Daily step count has been associated with cardiovascular disease, diabetes, and obesity, and is widely used in wearable studies and remote health monitoring [4,5,6,7,8]. Because these applications often rely on free-living wearable measurements, errors in step estimates can weaken downstream associations between daily steps and health outcomes.

Accurate step counting remains difficult outside controlled walking trials. Feito et al. [9] and Mora-Gonzalez et al. [10] showed that activity monitors can perform well during controlled walking, where gait is continuous and cadence is relatively stable. However, Toth et al. [11] reported substantially larger errors in video-recorded free-living validation. These results indicate that performance in clean periodic walking does not necessarily transfer to daily activity.

This difficulty arises from three sources of signal variation. First, walking may be interrupted by standing, turning, object interaction, and transitions between tasks, making steps less evenly spaced and less periodic [12]. Second, gait varies across people and walking speeds, with lower step-counting accuracy reported during slow walking and in older adults [13,14,15]. Third, sensor placement and non-walking motion introduce additional ambiguity, especially at the wrist, where arm motion can obscure true steps or produce step-like acceleration unrelated to walking [11,16].

Existing approaches address these sources of variation in different ways. Traditional step-counting methods rely on hand-designed signal rules, whereas deep learning methods learn more flexible representations from accelerometer data [16,17,18,19,20,21,22,23]. Large-scale self-supervised pretraining has also been applied to wearable sensing and free-living step counting [24,25]. Deep learning methods still require sufficient labeled target data, while self-supervised approaches rely on successful transfer from external data. Thus, methods that can achieve robust step counting using only labeled data from the target dataset remain necessary.

**Figure 1:**
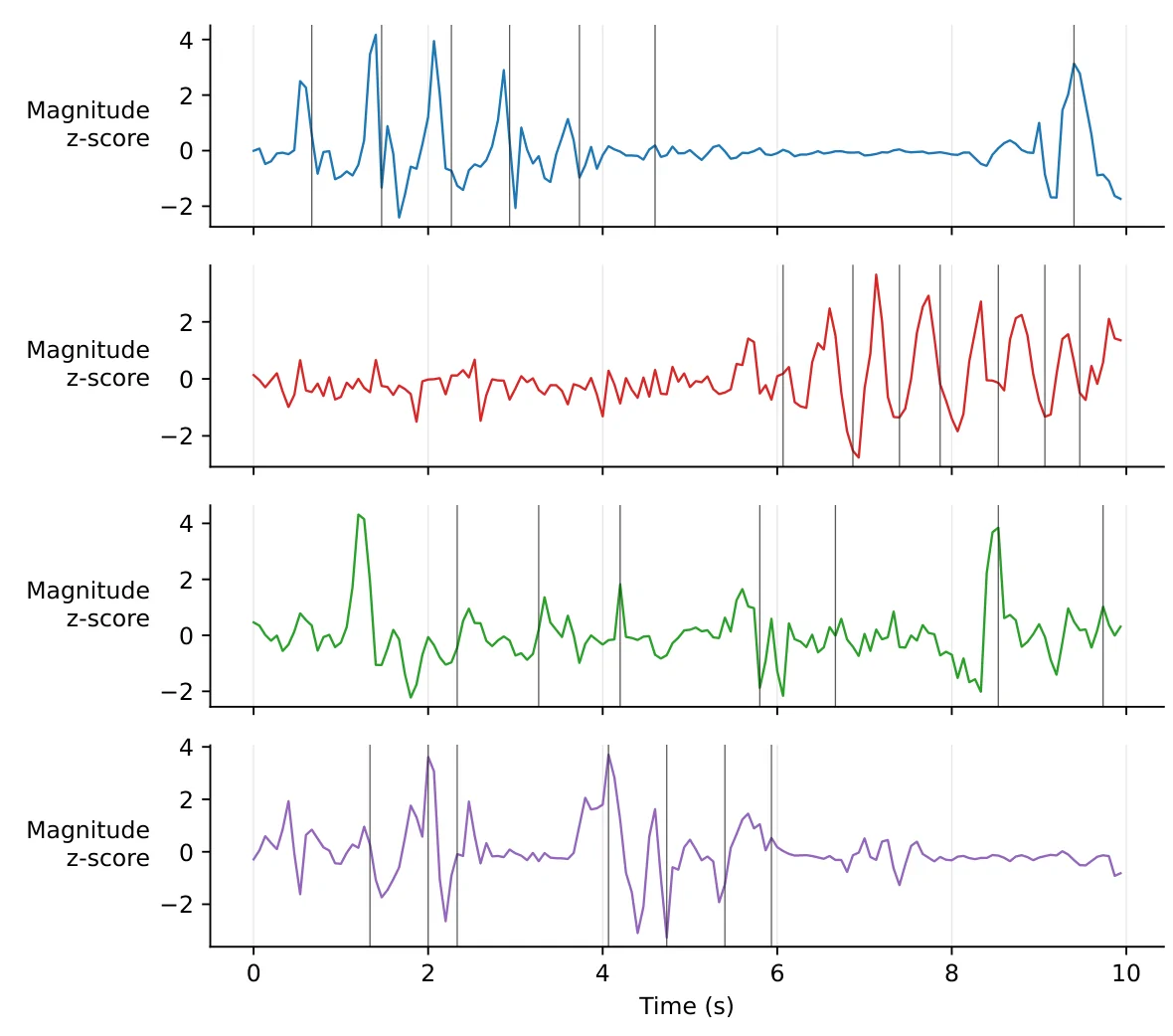
Examples of 10-s windows with the same count of 7 annotated steps but different temporal organization. Gray vertical lines mark the timing of the annotated steps.

The common issue across these sources of variation is that a given step count does not correspond to a consistent acceleration pattern. Figure 1 shows four 10-s windows with the same count of seven annotated steps but different temporal organization. The acceleration around an individual step recurs throughout the recording and is expected to be more stable than the pattern of an entire window, so individual steps form a more learnable target than a count over the whole window.

This motivates temporal step supervision. Each annotated step time is expanded into a short label interval, and a dense sequence model predicts step probability at every time step. The predicted probability is summed to estimate the count, so the model learns local step evidence before the window count is formed. In this study, we use a U-Net as the dense predictor, while the central contribution is the use of step timing annotations to supervise local step evidence before count aggregation.

The main contributions of this work are as follows:

- We formulate accelerometer step counting as a temporal supervision problem. Individual step times provide the training target, the model predicts step evidence over time, and the count is obtained by aggregating this evidence.
- We develop a training pipeline for this formulation, including dense label construction, a dense localization model, and a combined localization and count objective.
- We show that temporal step supervision improves count accuracy as walking departs from continuous periodic gait toward intermittent and uncontrolled movement, using only training data from the target dataset.
- We evaluate the framework across sensor locations and datasets, including wrist, hip, and ankle recordings and zero-shot transfer between Clemson and OxWalk. Without large-scale external pretraining, the framework achieves performance comparable to fine-tuned Stepcount SSL under irregular and free-living walking.

## 2. Related Work

### 2.1. Traditional Step-Counting Algorithms

Traditional step-counting methods usually detect steps from temporal or spectral patterns in the acceleration signal. Temporal methods include thresholding [26], peak detection [27], zero crossing, autocorrelation [28], and template matching, while spectral methods use Fourier or wavelet representations [12,16,17,18]. These methods are simple and efficient, but they depend on manually specified parameters such as thresholds, window lengths, periodicity assumptions, or frequency ranges. As a result, their performance can be sensitive to sensor placement, noise, and changes in the structure of the movement signal [3,12,13,14,29].

More recent signal-processing approaches include ADEPT for data-derived walking-stride segmentation [30], Oak/Forest for walking recognition using acceleration amplitude, periodicity, and duration criteria [31], and Verisense for wrist-accelerometer step counting [32].

### 2.2. Machine Learning and Deep Learning for Step Counting

Machine learning methods for step counting replace manually specified signal rules with learned mappings from inertial measurements to step estimates. Early learning approaches commonly used extracted inertial features with classifiers or regressors, while deep learning methods learn directly from raw acceleration signals. Chen [19] used an LSTM recurrent network for step counting, Shao et al. [20] used a convolutional network to suppress periodic negative-step motion, Pillai et al. [21] studied domain adaptation for personalized LSTM step counting, and Khan and Abedi [22] introduced an attention LSTM model for step-count regression. Although these models can learn flexible signal representations, their high capacity can make them prone to overfitting when labeled target data are limited.

Related work in adjacent sensing and vision domains has also used temporally structured representations for fine-grained recognition, including entropy-informed EEG modeling for driver-state recognition [33] and multi-scale temporal differencing for silhouette-based gait recognition [34].

### 2.3. Self-Supervised Learning for Wearable Sensing

One way to reduce dependence on labeled data is self-supervised learning. Large wrist accelerometer datasets provide substantial unlabeled data collected during daily life, allowing models to learn general signal representations before adaptation to downstream tasks. UK Biobank includes wrist accelerometer data from more than 100,000 participants [7,35], and Yuan et al. [24] used 700,000 person-days of wearable data for self-supervised human activity recognition. Small et al. [25] used large-scale self-supervised pretraining on UK Biobank wrist accelerometer data before fine-tuning on OxWalk for free-living step counting, achieving 12.5% MAPE. However, this approach still requires representations learned from external data to transfer to the target setting, and this transfer is not guaranteed when the source and target domains differ.

### 2.4. Step Counting Under Irregular and Free-Living Gait

Mattfeld et al. [12] showed that conventional pedometer algorithms degrade as gait becomes less regular, with reported error ranges of 3–8% for regular walking, 7–34% for semi-regular walking, and 11–466% for irregular walking. Toth et al. [11] showed that free-living step counting remains difficult, with wrist-worn devices routinely showing errors above 20% against camera-annotated ground truth. These findings highlight a remaining gap for step-counting methods that can preserve accuracy under irregular and free-living-like gait, where wrist acceleration signals are less periodic and less consistently aligned with steps.

## 3. Temporal Step Supervision Framework

### 3.1. Overview

**Figure 2:**
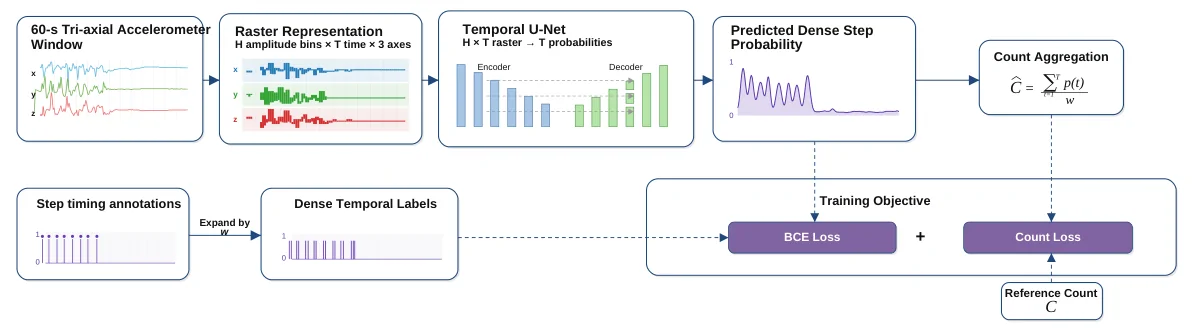
Overall architecture of the proposed temporal step supervision framework.

The proposed framework is shown in Figure 2. The key idea is to use annotated step times as temporal supervision during training. Unlike a conventional regression or forecasting framework that supervises the model only with a window count, temporal step supervision uses each step annotation to define where step evidence should appear in the input window. The model therefore learns a temporal step probability sequence, and the final count is obtained by summing this sequence over time.

Given a three-axis accelerometer window, we first convert it into an image-like time–amplitude raster representation for the U-Net. The raster input is passed to a U-Net predictor that preserves alignment with the input time samples. During training, dense temporal labels supervise step localization, and the reference count supervises the aggregated prediction.

### 3.2. Dense Temporal Labels

To train the model with temporal step information, each annotated step time is converted into a dense supervision target over nearby samples. The interval contains *w* samples centered at $t_{j}$: for odd *w*, samples

$$t_{j}-\left\lfloor w/2\right\rfloor ,\ldots ,t_{j}+\left\lfloor w/2\right\rfloor$$

are assigned label 1, inclusive. All other samples are assigned label 0.

This label construction preserves step timing information during training. A window count specifies how many steps occurred in the window, but it does not specify where they occurred. The dense labels provide this missing temporal information by assigning supervision to the local region around each annotated step.

### 3.3. Raster Representation

The raster representation is used to preserve temporal alignment while encoding local acceleration changes in an amplitude dimension. For raster inputs, each accelerometer axis is converted into a binary time–amplitude map. Given a tri-axial accelerometer window $X\in R^{3\times T}$, let $x_{c}\left(t\right)$ denote the value of axis *c* at time *t*. Each axis is clipped to a training-derived amplitude range $\left[v_{\min,c},v_{\max,c}\right]$ and quantized into *H* uniformly spaced bins:

$$b_{c}\left(t\right)=\left\lfloor \frac{\mathrm{clip}\left(x_{c}\left(t\right),v_{\min,c},v_{\max,c}\right)-v_{\min,c}}{v_{\max,c}-v_{\min,c}+\epsilon }\cdot \left(H-1\right)\right\rfloor ,$$

where ϵ prevents division by zero.

A vertical trace is drawn between consecutive bin indices $b_{c}\left(t\right)$ and $b_{c}\left(t+1\right)$ at columns *t* and t+1. The three axis maps are then stacked to form

$$X_{\mathrm{raster}}\in {\left\{0,1\right\}}^{3\times H\times T}.$$

### 3.4. Step Localization Model

Temporal step supervision requires a model whose outputs remain aligned with the input time samples. We therefore use a U-Net predictor [36] for dense temporal prediction. The encoder aggregates local signal patterns and broader context, while skip connections pass high-resolution encoder features to the decoder to retain timing information needed for short step intervals.

For each input window, the model outputs

$$\hat{p}\in {\left[0,1\right]}^{B\times T},$$

where ${\hat{p}}_{i}\left(t\right)$ denotes the predicted support for the dense step label at sample *t* in window *i*. The sequence is later aggregated to estimate the window count.

For raster inputs, the model receives

$$R\in R^{B\times 3\times H\times T},$$

where *B* is the batch size, *H* is the number of amplitude bins, and *T* is the number of time samples. Pooling is applied along the amplitude dimension, so the time dimension remains at length *T* throughout the network. The decoder restores the amplitude dimension, after which the amplitude axis is collapsed to form a temporal feature sequence. A final temporal prediction head maps this sequence to one output channel followed by a sigmoid activation.

The architectural configurations used in the experiments are reported in Appendix A.3.

### 3.5. Count Estimation and Training Loss

Each annotated step contributes approximately *w* positive samples to the dense target. The predicted count for window *i* is computed by summing the temporal probabilities and normalizing by the label width:

$${\hat{C}}_{i}=\frac{1}{w}\sum_{t=1}^{T}{\hat{p}}_{i}\left(t\right).$$

The reference count $C_{i}$ is the number of annotated steps in the same window.

The dense temporal prediction is trained with Binary Cross Entropy (BCE):

$$L_{\mathrm{BCE}}=-\frac{1}{BT}\sum_{i=1}^{B}\sum_{t=1}^{T}\left[y_{i}\left(t\right)\log{\hat{p}}_{i}\left(t\right)+\left(1-y_{i}\left(t\right)\right)\log\left(1-{\hat{p}}_{i}\left(t\right)\right)\right].$$

A count loss aligns the total predicted probability mass with the reference count. Within each mini-batch, windows from the same participant are aggregated before computing the count error. Let $I_{s}$ be the set of mini-batch windows from participant *s*. The aggregated predicted and reference counts are

$${\hat{C}}_{s}=\sum_{i\in I_{s}}{\hat{C}}_{i},\;C_{s}=\sum_{i\in I_{s}}C_{i}.$$

The count loss is

$$L_{\mathrm{count}}=\frac{1}{\left|S\right|}\sum_{s\in S}\frac{{\left({\hat{C}}_{s}-C_{s}\right)}^{2}}{C_{s}+1},$$

where S is the set of participants represented in the mini-batch.

The total objective at epoch *e* is

$$L_{\mathrm{total}}\left(e\right)=\left(1-\alpha_{e}\right)L_{\mathrm{BCE}}+\alpha_{e}L_{\mathrm{count}}.$$

The count weight follows a linear schedule:

$$\alpha_{e}=\left\{\begin{array}{cc} 0, & e<E_{\mathrm{warmup}},\\ \alpha_{\max}\frac{e-E_{\mathrm{warmup}}}{E_{\mathrm{ramp}}}, & E_{\mathrm{warmup}}\leq e<E_{\mathrm{warmup}}+E_{\mathrm{ramp}},\\ \alpha_{\max}, & e\geq E_{\mathrm{warmup}}+E_{\mathrm{ramp}}. \end{array}\right.$$

The schedule parameters are reported in Appendix A.4. This schedule first trains the model to localize step evidence from the dense labels, then gradually increases the influence of the count term so that the summed probability mass matches the window count.

## 4. Experiments

### 4.1. Datasets

We evaluate temporal step supervision on two accelerometer benchmarks with step annotations: Clemson and OxWalk.

Clemson contains data from 30 participants who completed three activity conditions designed to vary gait regularity [12]. Regular gait refers to structured walking under controlled conditions. Semi-regular gait refers to movement through buildings, where periods of regular walking are interrupted by brief stops, starts, or changes in direction. Irregular gait refers to the original unstructured gait condition in Clemson, where activity within a room consists mainly of short walking bouts of approximately 3–10 steps interrupted by rest or changes in direction. The recordings were sampled at 15 Hz and included wrist, hip, and ankle tri-axial accelerometer data. Synchronized video recordings were used to annotate step timing.

OxWalk was used to evaluate free-living step counting. It contains accelerometer recordings and camera-derived step annotations from 39 participants collected during natural daily activity [25]. We used the 25 Hz wrist and hip tri-axial accelerometer recordings.

### 4.2. Experimental Setup

Recordings were segmented into non-overlapping 60-s windows. Clemson windows contained T=900 samples, and OxWalk windows contained T=1500 samples. Final windows shorter than 60 seconds were zero-padded to the full length, and padded samples were excluded from label construction and count evaluation. Windows with no annotated steps were assigned a reference count of zero. Clemson recordings were windowed separately within each participant and walking condition, so windows did not cross regular, semi-regular, or irregular condition boundaries.

The proposed model was implemented in PyTorch version 2.5.1+cu121 [37] and trained using Adam [38]. The learning rate was $3\times 10^{-4}$, the weight decay was $1\times 10^{-4}$, and the batch size was 64. A cosine annealing learning-rate schedule was used with $\eta_{\min}=10^{-5}$, and gradients were clipped to norm 1.0. The maximum training budget was 120 epochs. Additional implementation details are provided in Appendix A.

For Clemson, all experiments used the same ten participant splits with a 70/20/10 training, validation, and test allocation. Each split contained 21 training participants, 6 validation participants, and 3 test participants. Across the ten splits, each of the 30 participants appeared exactly once in the test set. For trainable models, the validation participants were used for model selection.

For OxWalk, we used a subject-wise 10-fold evaluation. The 39 participants were assigned to ten test folds by round-robin allocation, producing nine folds with four test participants and one fold with three test participants. For each fold, the remaining participants were split at the participant level using an 80/20 training/validation split. This resulted in 28 training, seven validation, and four test participants in nine folds, and 29 training, seven validation, and three test participants in the remaining fold.

### 4.3. Baseline Methods

We compared the proposed method with three classical step-detection algorithms: peak detection, threshold crossing, and autocorrelation [12,26,27,28]. These methods detect steps from local peaks, threshold crossings, and periodicity in the acceleration magnitude signal. Parameters were selected from training data for each split and applied to the corresponding test participants.

CNN, WaveNet, and LSTM pipelines from prior Clemson step-counting work [23] were included as trainable neural comparisons. Appendix C reports their reproduction under the original Clemson evaluation protocol.

We also evaluated OxWearables Stepcount using its self-supervised pretrained encoder followed by supervised fine-tuning. On Clemson, wrist recordings were resampled from 15 Hz to 30 Hz to match the encoder input. For each train/validation/test split, Stepcount was fine-tuned using only the training subjects, while the validation subjects were used for early stopping and peak-count parameter selection. The same procedure was used on OxWalk, with the test subjects matched to those used in the main OxWalk experiment.

Recent step-counting methods included ADEPT [30], Oak/Forest [31], Verisense [32], and the public OxWearables Stepcount RF and SSL pipelines [25]. Their public implementations were evaluated on the same Clemson participant-condition units and OxWalk participants used for the corresponding comparison.

Following Rowlands et al. [32], we evaluated the original and revised Verisense parameterizations reported in that study. The original parameters were developed on the Clemson laboratory gait dataset, whereas the revised parameters were optimized on a 16-participant subset of a separate free-living dataset.

### 4.4. Experimental Comparisons

We evaluated the contribution of temporal supervision by comparing four training objectives for the temporal U-Net: count loss only, binary cross entropy (BCE) only, BCE + count loss with no schedule, and BCE + count loss with the scheduled count term.

Clemson used gravity-removed raster input with the original training annotations. OxWalk used unprocessed raster input with motion-energy realigned training labels. The complete preprocessing and label-construction comparison is reported in Appendix E.

We evaluated temporal step supervision across the sensor locations available in each dataset. Clemson included wrist, hip, and ankle recordings, while OxWalk included wrist and hip recordings. For each sensor location, we compared 1D sequence and raster inputs using unprocessed and gravity-removed acceleration. Models were trained separately for each sensor location. Motion-energy realigned labels were used for the Clemson comparison, while both original and realigned training labels were evaluated on OxWalk.

We evaluated sensitivity to dense-label width using widths of 1, 3, 5, 7, 9, and 11 samples for Clemson and 1, 3, 5, 7, 9, 11, and 31 samples for OxWalk. The main label widths were set to 5 samples for Clemson and 7 samples for OxWalk, corresponding to approximately one third of a second in both datasets, which is comparable to the duration of a single step. Clemson retained the original training annotations because the controlled laboratory recordings provided relatively consistent alignment between annotated step times and the accelerometer response. In contrast, motion-energy realignment was used for the more variable free-living OxWalk recordings to reduce timing mismatch between camera-derived step annotations and local accelerometer peaks (Appendix A.6).

Zero-shot transfer was evaluated in both directions between Clemson and OxWalk using wrist recordings. OxWalk was resampled from 25 Hz to 15 Hz, and the dense-label width was set to 5 samples. Models were trained on the source dataset and evaluated directly on the target dataset. OxWalk-to-Clemson performance was reported for regular, semi-regular, and irregular walking, while Clemson-to-OxWalk performance was aggregated by participant. Matched in-domain reference results were also obtained for temporal step supervision and Stepcount SSL using OxWalk recordings resampled to 15 Hz, matching the target-data sampling condition used in the Clemson-to-OxWalk transfer evaluation.

### 4.5. Evaluation Metrics

For Clemson, predicted and reference counts were summed for each participant and gait condition. For OxWalk, counts were summed across free-living windows for each participant. Let ${\hat{C}}_{s}$ and $C_{s}$ denote the predicted and reference counts for evaluation unit *s*.

Absolute percentage error was computed as

$${\mathrm{APE}}_{s}=100\left|\frac{{\hat{C}}_{s}-C_{s}}{C_{s}}\right|,$$

and mean absolute percentage error (MAPE) was calculated as the mean APE. Clemson MAPE was reported by gait condition and overall across the 90 participant-condition observations. OxWalk MAPE was computed across participants.

Percentage error was computed as

$${\mathrm{PE}}_{s}=100\frac{{\hat{C}}_{s}-C_{s}}{C_{s}},$$

and mean percentage error (MPE) quantifies counting bias.

Uncertainty in MAPE and MPE was quantified using 95% bootstrap confidence intervals based on 10,000 bootstrap resamples. Participants were resampled with replacement within each dataset, and both metrics were recomputed for each bootstrap sample. For Clemson overall confidence intervals, participants were resampled with all three gait-condition results from each sampled participant kept together. Point estimates were computed using all evaluation units, while confidence intervals were obtained from the bootstrap resamples.

Improvement was assessed using lower MAPE, MPE closer to zero, and smaller increases in error as gait became less regular. Robustness was assessed from changes in MAPE and MPE across Clemson gait conditions.

## 5. Results

### 5.1. Comparison with Deep Learning Baselines

Table 1 and Table 2 compare temporal step supervision with CNN, WaveNet, LSTM, and fine-tuned OxWearables Stepcount SSL on Clemson wrist recordings.

The largest differences between methods appeared under irregular walking. Temporal step supervision achieved MAPE values of 4.61%, 9.90%, and 15.78% under regular, semi-regular, and irregular walking, respectively. CNN, WaveNet, and LSTM achieved low error under regular walking, but their irregular-walking MAPE increased to 129.77%, 263.02%, and 247.82%, respectively. Fine-tuned Stepcount SSL achieved MAPE values of 4.89%, 4.63%, and 14.42% across the three conditions. Thus, under irregular walking, temporal step supervision substantially outperformed the CNN, WaveNet, and LSTM baselines and achieved MAPE comparable to fine-tuned Stepcount SSL.

The bias analysis showed a different error pattern across methods. Temporal step supervision had an overall MPE of −1.58%, indicating little systematic bias across the three gait conditions. CNN, WaveNet, and LSTM showed uniform overcounting under irregular walking; consequently, their irregular-condition MPE values were equal to their corresponding MAPE values. Fine-tuned Stepcount SSL also showed little overall systematic bias, with an MPE of −0.73%.

On OxWalk, temporal step supervision achieved 13.06% MAPE and −2.78% MPE, compared with 11.07% MAPE and −3.50% MPE for fine-tuned Stepcount SSL (Table 3).

Paired comparisons of participant-level APE supported the same pattern. On Clemson, temporal step supervision did not differ significantly from fine-tuned Stepcount SSL (paired *t*-test p=0.29; Cohen’s $d_{z}=-0.20$), while its APE was lower than CNN, WaveNet, and LSTM (paired *t*-test p<0.01; $d_{z}=1.64$–3.34). On OxWalk, temporal step supervision and fine-tuned Stepcount SSL also showed no significant difference (paired *t*-test p=0.23; $d_{z}=-0.19$).

**Figure 3:**
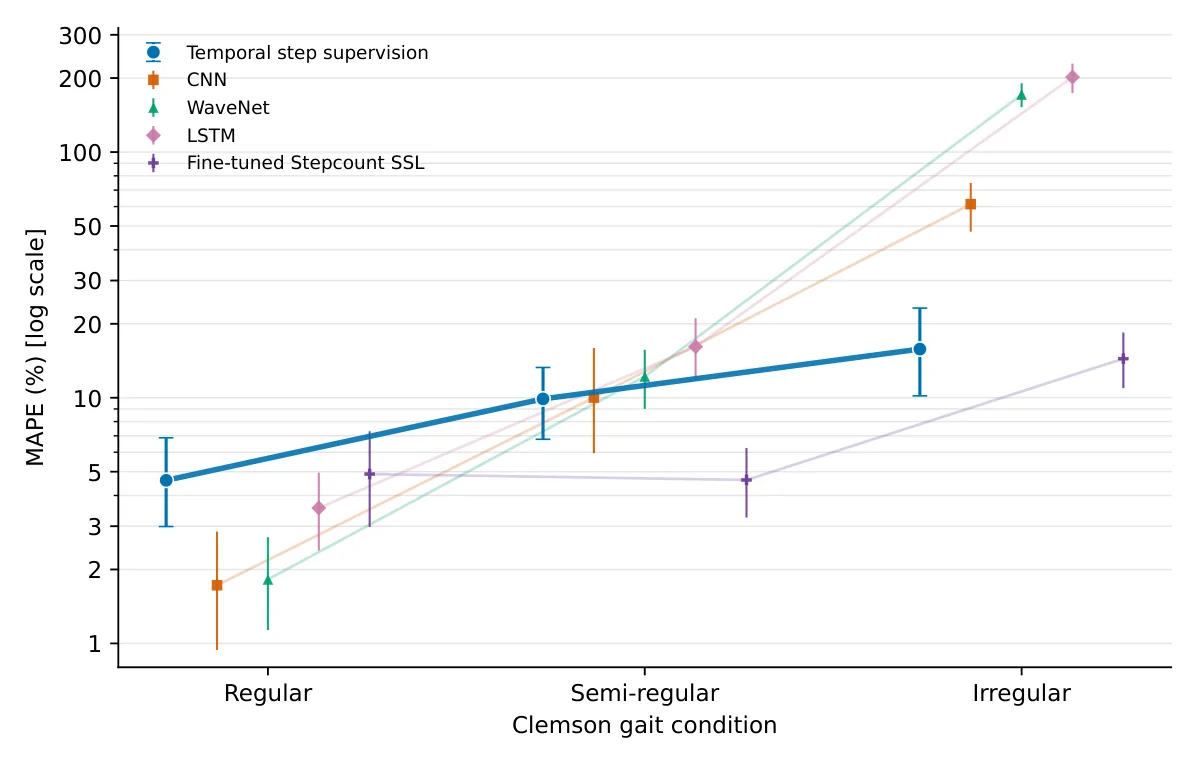
MAPE comparison of temporal step supervision and deep-learning baselines across Clemson gait conditions. Points indicate MAPE and error bars indicate 95% bootstrap confidence intervals. The vertical axis uses a logarithmic scale.

Figure 3 provides a visual summary of the MAPE comparison across Clemson gait conditions.

### 5.2. Comparison with Classical and Recent Step-Counting Methods

Table 4 and Table 5 compare temporal step supervision with classical and recent step-counting methods on Clemson. Autocorrelation and peak detection performed well under regular walking, but their errors increased as gait became less regular. Temporal step supervision achieved the lowest MAPE among the compared methods under both semi-regular and irregular walking. Verisense original was the next-best method under irregular walking, followed by Oak/Forest and autocorrelation. The public Stepcount SSL pipeline showed substantially higher error under Clemson irregular walking, reaching 99.18% MAPE. This may partly reflect a mismatch between its original operating conditions and the Clemson data: the pipeline was developed for 30 Hz free-living wrist recordings, whereas Clemson was recorded at 15 Hz and includes short interrupted walking bouts. Its walking-detection stage may therefore reject some of these brief bouts. The substantially lower error obtained after supervised fine-tuning on Clemson supports the interpretation that this result reflects domain and pipeline mismatch rather than a general limitation of the underlying representation.

The MPE results showed different error patterns across methods. Several public pipelines systematically undercounted, whereas peak and threshold detection strongly overcounted under irregular walking. Temporal step supervision showed little overall systematic bias.

On OxWalk, temporal step supervision achieved the lowest MAPE among the classical and public pipelines in Table 6, followed by autocorrelation and the OxWearables stepcount RF model.

### 5.3. Loss Objective Comparison

Table 7 and Table 8 compare the four training objectives on Clemson. Removing the loss schedule produced only a modest increase in overall MAPE. In contrast, removing either component of the hybrid objective caused substantially larger degradation under irregular walking. BCE-only training increased irregular-gait MAPE to 22.32%, while count-only training increased it to 46.87%. Their corresponding overall MAPE values were 12.59% and 22.60%.

The same pattern was observed on OxWalk (Table 9). BCE-only and count-only training produced MAPE values of 22.70% and 53.30%, respectively, while removing the schedule resulted in 23.68% MAPE. The scheduled hybrid objective therefore provided the most consistent performance across the two datasets.

### 5.4. Dense-Label Width Sensitivity

Table 10 shows the effect of dense-label width on Clemson. Performance was relatively stable for intermediate widths. Widths 3, 5, and 7 achieved overall MAPE values of 10.33%, 10.10%, and 10.12%, respectively. In contrast, width 1 increased overall MAPE to 17.79%, while width 11 increased it to 20.47%. The deterioration at the widest setting was especially pronounced under regular walking.

A similar pattern was observed on OxWalk (Table 11). Widths from 5 to 11 produced MAPE values between 11.05% and 13.06%, whereas width 1 increased MAPE to 24.42% and width 31 increased it to 42.09%. These results indicate that performance was robust over a range of intermediate label widths but degraded when temporal supervision became either very narrow or excessively broad.

### 5.5. Across Sensor Locations and Input Configurations

Table 12, Table 13 and Table 14 compare sensor locations, input representations, acceleration preprocessing, and, for OxWalk, training-label configurations. For Clemson, this comparison uses motion-energy-realigned training labels; therefore, the 11.13% wrist raster result with gravity removal differs from the 10.10% main result obtained with the original training annotations.

On Clemson, the best-performing configuration varied by sensor location. The lowest MAPE was 11.10% at the wrist with 1D input and gravity removal, 8.86% at the hip with raster input and unprocessed acceleration, and 7.90% at the ankle with 1D input and gravity removal. The corresponding MPE values were −3.51%, −1.49%, and −0.58%. Across the tested configurations, hip and ankle recordings generally produced lower error than wrist recordings.

On OxWalk, the lowest hip MAPE was 6.73% with raster input, gravity removal, and realigned training labels. At the wrist, the main unprocessed raster configuration with realigned training labels achieved 13.06% MAPE. The corresponding unprocessed raster configuration with original labels achieved 14.07%, while gravity removal with realigned labels achieved 16.25%. These results show that performance depended on sensor location, input representation, preprocessing, and training-label configuration.

### 5.6. Zero-Shot Cross-Dataset Generalization

Table 15 and Table 16 show transfer from OxWalk to Clemson. Temporal step supervision achieved an overall MAPE of 14.19% and remained relatively stable across gait conditions. Autocorrelation performed particularly well under regular walking at 0.68% MAPE, but increased to 50.46% under irregular walking. Stepcount SSL achieved an overall MAPE of 9.79%, while temporal step supervision showed less variation in error across the three gait conditions.

Table 17 shows transfer in the opposite direction, from Clemson to OxWalk. On OxWalk, the Clemson-trained temporal step supervision model achieved 28.75% MAPE, compared with 17.28% for autocorrelation and 24.85% for Stepcount SSL. Peak and threshold detection produced substantially larger errors of 122.22% and 791.55%, respectively.

To distinguish cross-dataset transfer effects from the effect of the 15 Hz target-data sampling condition, Table 18 compares the two learned methods with matched 15 Hz in-domain references. Temporal step supervision increased from 17.38% to 28.75% MAPE, while Stepcount SSL increased from 13.96% to 24.85%, corresponding to increases of 11.37 and 10.89 percentage points, respectively. These results show an asymmetric cross-dataset transfer pattern and indicate that both learned methods were affected by the Clemson-to-OxWalk domain shift.

### 5.7. Computational Cost

Table 19 compares model size and CPU inference time for temporal step supervision and the deep-learning baselines. Temporal step supervision contained 1.05 million trainable parameters, compared with 0.11 million for CNN, 0.02 million for WaveNet, 0.21 million for LSTM, and 10.98 million for fine-tuned Stepcount SSL.

Processing a 60 s recording required 86.91±2.15 ms with temporal step supervision. This was faster than LSTM (203.54±3.07 ms), but slower than CNN (12.95±0.68 ms), WaveNet (46.03±2.01 ms), and fine-tuned Stepcount SSL (48.23±0.94 ms). All timings were measured on CPU using one intra-op and one inter-op thread, with batch size 1, after 20 warm-up runs and over 100 timed repeats. Because the models use different native input formats, each row reports the complete inference pipeline required to process an equivalent 60-s recording. Additional pipeline-specific details are provided in Appendix B.

## 6. Discussion

This study evaluated temporal step supervision for accelerometer step counting under structured, irregular, and free-living conditions. The central result is that using step timing annotations during training improved count estimation when walking became less regular. The comparison with classical detectors shows that this advantage emerges mainly under irregular gait. Previous step-counting work commonly trains models from aggregate counts, which state how many steps occur in a window but not where the evidence for those steps appears. This limits the training signal when gait contains pauses, turns, changing cadence, and uneven spacing. The proposed temporal supervision addresses this by using binary cross-entropy loss to constrain the model output near annotated step times, while the count term calibrates the total probability mass against the reference count.

The Clemson results show that regular walking performance alone is not sufficient to judge robustness. The classical algorithms and reproduced CNN, WaveNet, and LSTM pipelines performed well during regular walking but generally degraded under irregular walking. This pattern is consistent with the structure of the Clemson irregular condition, where short walking bouts are interrupted by pauses and changes in direction. Although LSTMs are a natural choice for accelerometer time-series modeling, this interrupted structure may reduce the regular temporal continuity that recurrent models can exploit in sustained walking. Temporal supervision addresses this limitation by directing the model toward localized signal evidence around annotated step times, which is more consistent than the global temporal continuity of the full walking sequence under interrupted walking conditions. Across Clemson and OxWalk, temporal step supervision showed competitive MAPE and overall MPE close to zero, indicating that its counting error was not accompanied by strong systematic overcounting or undercounting.

The loss ablation results further support the use of both components of the training objective. The substantial increase in error when either component was used alone shows that temporal localization and count calibration are complementary, particularly under irregular and free-living walking. The dense-label experiments also showed that performance was relatively stable across moderate label widths, while very narrow or very broad labels degraded performance. The stronger settings corresponded approximately to the temporal extent of a single step, suggesting that the dense target should represent the local step event rather than only the annotated time point.

Prior OxWalk work used large-scale self-supervised pretraining on UK Biobank before fine-tuning on OxWalk [25]. Temporal step supervision achieved performance similar to fine-tuned Stepcount SSL under irregular and free-living walking, despite being trained only on the target datasets without large-scale external pretraining. This result suggests that temporal step supervision can reduce dependence on external pretraining. This does not rule out further gains from combining temporal step supervision with large unlabeled accelerometer datasets.

The cross-dataset experiments showed an asymmetric transfer pattern. Temporal step supervision transferred more effectively from OxWalk to Clemson than from Clemson to OxWalk. In the OxWalk-to-Clemson direction, its error remained comparatively stable across regular, semi-regular, and irregular walking. Stepcount SSL achieved lower overall error in this direction. In the Clemson-to-OxWalk direction, temporal step supervision increased from 17.38% matched in-domain MAPE to 28.75% under zero-shot transfer, while Stepcount SSL increased from 13.96% to 24.85%, corresponding to similar increases of 11.37 and 10.89 percentage points, respectively. This similar degradation across the two learned methods supports the interpretation that both were affected by the domain shift from the structured Clemson recordings to the free-living OxWalk setting. Autocorrelation achieved lower error than both learned methods in this direction. Although temporal supervision reduces dependence on large-scale training data, it remains a deep learning model and still depends on sufficient variability in the training data. The structured Clemson recordings may therefore provide less diverse training information for transfer to free-living OxWalk conditions. This difference is also reflected in the annotation-derived timing statistics in Appendix D, which show more fragmented temporal step patterns in OxWalk than in Clemson. Autocorrelation, in contrast, does not rely on training data and may be less sensitive to this limitation. This asymmetry shows that reducing training-data requirements does not eliminate deep learning models’ dependence on training-data diversity.

The preprocessing and label construction experiments indicate that input representation and label timing both affect performance, but their effects appear to depend on the structure of the recorded movement signal. Performance also varied across sensor locations, with hip and ankle recordings generally producing lower error than wrist recordings, likely because they more directly capture gait-related motion. These results suggest that preprocessing should not be treated as a fixed default: rasterization, gravity removal, and motion-energy realignment each helped in different settings depending on sensor location and movement structure.

From a computational perspective, temporal step supervision used substantially fewer parameters than fine-tuned Stepcount SSL and processed a 60s recording in less than 100ms in the CPU benchmark. Its inference time was higher than CNN, WaveNet, and Stepcount SSL but lower than LSTM. These results suggest that the model is practical for offline or near-real-time analysis.

### Limitations and Future Directions

Temporal step supervision requires annotations that identify when individual steps occur. This is an inherent limitation of the method, as obtaining step-level timing annotations is more demanding than collecting aggregate step counts alone. This requirement also limits the availability of suitable public wearable datasets. Consequently, the cross-dataset evaluation in this study was limited to wrist recordings from two datasets. Public datasets providing raw accelerometer signals with synchronized step annotations remain particularly limited for clinical populations and controlled variations in gait patterns, sensor orientation, and device type. Future work should therefore evaluate the robustness and generalizability of temporal step supervision on pathological and atypical gait, additional datasets, and broader sensor configurations.

Embedded deployment also requires further evaluation. The current inference benchmark was conducted on a CPU platform, while the raster representation expands the input from 3×T to 3×16×T, increasing memory requirements for resource-constrained devices.

More detailed annotations that mark the start and end of each step could also support temporal event detection. The current method uses a fixed temporal region around each annotated step time because the available annotations specify the time point of each step but not its full duration. If step onset and offset were available, the temporal extent of each step could instead be learned directly rather than defined using a fixed label width. In that setting, YOLO-style detectors could be adapted to time–amplitude raster inputs, with each step represented by a predicted temporal interval and confidence score before aggregation into a count.

## 7. Conclusions

This study shows that step-counting performance can be improved by using annotated step data and a model architecture that accounts for temporal information. Temporal step supervision preserved step timing information, and the hybrid BCE and count loss objective linked local step evidence to the final count. These findings suggest that careful construction of supervision targets, model outputs, and loss functions can be an effective alternative to relying only on larger models or larger external pretraining datasets.

## Figures and Tables

**Table 1:** MAPE comparison with deep learning baselines on Clemson wrist recordings. Values are percentages with 95% bootstrap confidence intervals in brackets. The lowest MAPE in each column is shown in bold.

Method	Regular	Semi-Regular	Irregular	Overall
CNN	1.64[0.93,2.56]	11.43[8.41,14.71]	129.77[109.99,150.80]	47.61[40.81,54.95]
WaveNet	1.92[1.36,2.54]	20.86[17.30,24.65]	263.02[238.18,287.90]	95.27[86.56,104.01]
LSTM	4.32[2.64,6.33]	17.02[13.23,21.15]	247.82[219.98,273.79]	89.72[80.67,98.44]
Fine-tuned Stepcount SSL	4.89[2.98,7.24]	4.63[3.21,6.26]	14.42[10.88,18.43]	7.98[6.24,9.92]
Temporal step supervision	4.61[2.98,6.99]	9.90[6.80,13.22]	15.78[10.11,23.20]	10.10[7.30,13.66]

**Table 2:** MPE comparison with deep learning baselines on Clemson wrist recordings. Values are percentages with 95% bootstrap confidence intervals in brackets. The MPE closest to zero in each column is shown in bold.

Method	Regular	Semi-Regular	Irregular	Overall
CNN	1.54[0.80,2.46]	8.04[3.49,12.05]	129.77[109.99,150.76]	46.45[38.97,54.25]
WaveNet	1.58[0.89,2.29]	19.12[14.32,23.72]	263.02[238.80,287.98]	94.57[85.58,103.95]
LSTM	3.37[1.36,5.62]	14.28[9.00,19.21]	247.82[220.57,273.88]	88.49[78.76,97.82]
Fine-tuned Stepcount SSL	−4.89[−7.24,−2.98]	1.14[−1.12,3.33]	1.55[−4.77,8.19]	−0.73[−3.64,2.33]
Temporal step supervision	−2.33[−5.10,−0.13]	−5.28[−9.55,−0.62]	2.87[−5.07,12.44]	−1.58[−5.69,3.12]

**Table 3:** Comparison with fine-tuned OxWearables Stepcount SSL on OxWalk wrist recordings. Values are percentages with 95% bootstrap confidence intervals in brackets. The lowest MAPE and the MPE closest to zero are shown in bold.

Method	MAPE	MPE
Fine-tuned Stepcount SSL	11.07[8.51,13.91]	−3.50[−7.69,0.79]
Temporal step supervision	13.06[9.79,16.58]	−2.78[−7.95,2.60]

**Table 4:** MAPE comparison with classical and recent step-counting methods on Clemson wrist recordings. Values are percentages with 95% bootstrap confidence intervals in brackets. The lowest MAPE in each column is shown in bold.

Method	Regular	Semi-Regular	Irregular	Overall
Peak detector	2.32[1.09,3.84]	18.92[13.95,24.82]	248.93[218.78,280.93]	90.06[78.84,102.40]
Threshold detector	6.55[3.76,9.89]	22.29[17.17,28.38]	382.65[347.29,424.71]	137.16[123.98,153.44]
Autocorrelation detector	1.76[0.52,4.03]	17.00[12.67,21.47]	51.70[44.60,58.84]	23.49[20.13,26.75]
ADEPT	26.64[21.63,32.38]	60.74[57.58,63.86]	81.74[78.67,84.82]	56.37[54.43,58.46]
Oak/Forest	18.56[10.49,27.76]	21.19[17.14,25.28]	33.62[24.29,44.24]	24.46[20.03,29.51]
Verisense (original)	16.66[12.74,20.80]	30.02[25.93,34.12]	22.91[17.65,28.77]	23.20[19.99,26.63]
Verisense (revised)	10.43[7.48,13.67]	29.59[24.72,34.57]	46.91[39.57,53.83]	28.98[25.08,32.92]
OxWearables Stepcount RF	6.33[4.80,8.23]	12.52[8.73,17.69]	63.83[55.07,71.86]	27.56[24.48,30.42]
OxWearables Stepcount SSL (public pipeline)	58.82[42.38,74.46]	53.72[38.68,68.71]	$99.18^{†}\;\left[98.19,\;100.00\right]$	70.57[63.48,77.64]
Temporal step supervision	4.61[2.98,6.99]	9.90[6.80,13.22]	15.78[10.11,23.20]	10.10[7.30,13.66]

^†^ The public Stepcount SSL pipeline was developed for 30 Hz free-living wrist data; mismatch with the 15 Hz Clemson recordings and short walking bouts may contribute to the high error, which was substantially reduced after supervised fine-tuning on Clemson.

**Table 5:** MPE comparison with classical and recent step-counting methods on Clemson wrist recordings. Values are percentages with 95% bootstrap confidence intervals in brackets. The MPE closest to zero in each column is shown in bold.

Method	Regular	Semi-Regular	Irregular	Overall
Peak detector	−1.86[−3.45,−0.56]	18.60[13.40,24.79]	248.93[218.61,281.00]	88.56[77.33,100.74]
Threshold detector	−5.81[−9.33,−2.84]	22.29[17.06,28.39]	382.65[347.31,426.06]	133.04[119.87,148.19]
Autocorrelation detector	−0.89[−3.25,0.46]	−13.06[−18.88,−7.31]	−37.85[−51.27,−22.72]	−17.26[−23.27,−10.50]
ADEPT	−26.64[−32.29,−21.56]	−60.74[−63.87,−57.62]	−81.74[−84.82,−78.59]	−56.37[−58.46,−54.47]
Oak/Forest	−18.38[−27.51,−10.11]	−18.49[−23.77,−12.66]	33.62[24.32,44.28]	−1.08[−6.49,4.17]
Verisense (original)	−16.66[−20.81,−12.80]	−29.76[−33.98,−25.54]	−12.78[−20.81,−3.11]	−19.73[−24.04,−15.23]
Verisense (revised)	−10.43[−13.65,−7.49]	−28.60[−34.05,−23.03]	−44.11[−52.81,−34.36]	−27.71[−32.38,−22.97]
OxWearables Stepcount RF	−6.33[−8.19,−4.78]	−0.28[−6.06,6.64]	−63.83[−71.64,−54.73]	−23.48[−27.64,−18.88]
OxWearables Stepcount SSL (public pipeline)	−58.82[−74.72,−42.41]	−52.68[−68.39,−36.83]	−99.18[−100.00,−98.23]	−70.23[−77.09,−63.33]
Temporal step supervision	−2.33[−5.10,−0.13]	−5.28[−9.55,−0.62]	2.87[−5.07,12.44]	−1.58[−5.69,3.12]

**Table 6:** MAPE and MPE comparison with classical and recent step-counting methods on OxWalk wrist recordings. Values are percentages with 95% bootstrap confidence intervals in brackets. The lowest MAPE and the MPE closest to zero are shown in bold.

Method	MAPE	MPE
Peak detector	32.26[24.72,41.77]	−5.55[−17.53,8.67]
Threshold detector	230.94[158.61,313.56]	230.37[155.72,314.45]
Autocorrelation detector	16.90[11.76,23.92]	−7.27[−13.91,1.61]
ADEPT	60.95[55.70,66.12]	−60.95[−66.19,−55.73]
Oak/Forest	25.83[14.09,40.76]	10.59[−3.11,27.15]
Verisense (original)	28.62[21.11,38.12]	−12.56[−22.77,0.52]
Verisense (revised)	33.03[26.49,40.77]	−25.34[−33.97,−14.17]
OxWearables Stepcount RF	21.34[11.57,36.81]	10.33[−1.20,26.50]
OxWearables Stepcount SSL (public pipeline)	31.77[21.99,42.60]	−29.98[−41.41,−19.55]
Temporal step supervision	13.06[9.79,16.58]	−2.78[−7.95,2.60]

**Table 7:** MAPE comparison of training objectives on Clemson wrist recordings. Values are percentages with 95% bootstrap confidence intervals in brackets. The lowest MAPE in each column is shown in bold.

Objective	Regular	Semi-Regular	Irregular	Overall
BCE + count, scheduled	4.61[2.98,6.99]	9.90[6.80,13.22]	15.78[10.11,23.20]	10.10[7.30,13.66]
BCE + count, no schedule	3.76[2.78,4.80]	10.62[7.47,14.38]	17.31[11.86,24.90]	10.56[8.15,13.89]
BCE only	4.76[3.48,6.21]	10.70[7.58,14.03]	22.32[15.46,31.36]	12.59[9.98,16.28]
Count only	6.96[4.92,9.41]	13.96[11.27,16.73]	46.87[37.65,57.57]	22.60[19.52,26.30]

**Table 8:** MPE comparison of training objectives on Clemson wrist recordings. Values are percentages with 95% bootstrap confidence intervals in brackets. The MPE closest to zero in each column is shown in bold.

Objective	Regular	Semi-Regular	Irregular	Overall
BCE + count, scheduled	−2.33[−5.10,−0.13]	−5.28[−9.55,−0.62]	2.87[−5.07,12.44]	−1.58[−5.69,3.12]
BCE + count, no schedule	−2.24[−3.67,−0.69]	−4.77[−9.48,0.20]	4.54[−3.46,14.32]	−0.82[−4.75,3.82]
BCE only	−2.65[−4.68,−0.68]	−7.01[−11.05,−2.44]	21.25[13.89,30.65]	3.86[0.24,8.28]
Count only	−5.36[−8.26,−2.79]	−12.06[−15.61,−8.22]	46.87[37.65,57.57]	9.82[5.78,14.21]

**Table 9:** Training-objective comparison on OxWalk wrist recordings. Values are percentages with 95% bootstrap confidence intervals in brackets. The lowest MAPE and the MPE closest to zero are shown in bold.

Objective	MAPE	MPE
BCE + count, scheduled	13.06[9.79,16.58]	−2.78[−7.95,2.60]
BCE + count, no schedule	23.68[18.47,29.30]	−11.31[−19.86,−2.67]
BCE only	22.70[16.15,31.07]	10.19[1.00,20.58]
Count only	53.30[33.74,76.60]	43.92[22.44,69.39]

**Table 10:** Dense-label width sensitivity on Clemson wrist recordings. Values are MAPE percentages with 95% bootstrap confidence intervals in brackets. The lowest value in each column is shown in bold.

Width	Regular	Semi-Regular	Irregular	Overall
1	7.01[4.97,9.30]	12.12[8.67,16.06]	34.26[16.40,56.28]	17.79[11.12,25.92]
3	5.16[3.43,7.48]	10.72[7.78,13.93]	15.11[9.88,22.09]	10.33[7.84,13.46]
5	4.61[2.98,6.99]	9.90[6.80,13.22]	15.78[10.11,23.20]	10.10[7.30,13.66]
7	3.81[2.35,5.80]	9.97[7.04,13.12]	16.57[11.34,23.71]	10.12[7.60,13.27]
9	12.63[11.02,14.21]	10.03[7.04,13.25]	15.38[9.62,23.15]	12.68[10.27,15.97]
11	27.96[26.40,29.45]	17.03[13.74,20.44]	16.43[9.02,27.16]	20.47[17.44,24.56]

**Table 11:** Dense-label width sensitivity on OxWalk wrist recordings. Values are percentages with 95% bootstrap confidence intervals in brackets. The lowest MAPE and MPE closest to zero are shown in bold.

Width	MAPE	MPE
1	24.42[19.52,30.01]	−14.00[−21.97,−5.77]
3	17.65[13.27,22.72]	−6.60[−13.79,0.34]
5	12.78[8.87,16.98]	−5.34[−10.69,0.19]
7	13.06[9.79,16.58]	−2.78[−7.95,2.60]
9	12.98[9.80,16.50]	−2.31[−7.50,2.83]
11	11.05[7.83,14.57]	−4.16[−8.71,0.45]
31	42.09[36.29,47.89]	−32.18[−41.42,−20.63]

**Table 12:** Sensor-location and input-configuration MAPE comparison on Clemson using motion-energy realigned training labels. Values are percentages with 95% bootstrap confidence intervals in brackets. Within each sensor location, the lowest MAPE in each column is shown in bold.

Position	Input	Preprocessing	Regular	Semi-Regular	Irregular	Overall
Ankle	Raster	Gravity removal	3.70[2.64,4.94]	8.34[4.63,12.80]	12.29[8.45,17.12]	8.11[5.66,11.21]
Ankle	Raster	Unprocessed	3.92[2.67,5.39]	8.40[5.02,12.89]	12.34[8.32,17.06]	8.22[5.86,11.25]
Ankle	1D	Gravity removal	4.56[3.23,6.12]	8.56[5.22,12.96]	10.59[6.93,15.25]	7.90[5.61,10.96]
Ankle	1D	Unprocessed	5.62[4.04,7.36]	9.14[6.06,13.24]	11.11[7.62,15.66]	8.62[6.45,11.47]
Hip	Raster	Gravity removal	3.83[2.80,5.01]	9.78[6.45,13.75]	13.49[9.56,18.31]	9.03[6.80,11.81]
Hip	Raster	Unprocessed	3.13[2.16,4.16]	9.75[6.55,13.69]	13.70[8.60,20.03]	8.86[6.50,11.85]
Hip	1D	Gravity removal	4.52[3.12,6.10]	8.56[5.32,12.50]	14.39[10.48,19.23]	9.16[6.84,11.98]
Hip	1D	Unprocessed	4.69[3.23,6.36]	10.59[5.34,17.86]	23.20[14.95,32.80]	12.83[8.51,18.17]
Wrist	Raster	Gravity removal	5.57[3.84,7.82]	9.90[6.88,13.30]	17.92[11.96,26.06]	11.13[8.29,14.73]
Wrist	Raster	Unprocessed	8.94[4.62,15.24]	15.69[10.65,22.15]	23.90[16.85,31.19]	16.18[11.74,21.71]
Wrist	1D	Gravity removal	6.20[4.28,8.31]	10.50[7.81,13.40]	16.59[12.02,21.94]	11.10[8.59,13.93]
Wrist	1D	Unprocessed	10.25[5.82,16.08]	17.67[12.31,23.69]	19.55[14.37,24.93]	15.82[11.93,20.53]

**Table 13:** Sensor-location and input-configuration MPE comparison on Clemson using motion-energy realigned training labels. Values are percentages with 95% bootstrap confidence intervals in brackets. Within each sensor location, the MPE closest to zero in each column is shown in bold.

Position	Input	Preprocessing	Regular	Semi-Regular	Irregular	Overall
Ankle	Raster	Gravity removal	−2.54[−4.12,−1.09]	−1.56[−6.17,3.88]	4.29[−1.37,10.66]	0.06[−3.48,4.16]
Ankle	Raster	Unprocessed	−2.87[−4.62,−1.26]	−1.62[−5.83,3.81]	6.16[0.46,12.41]	0.55[−2.85,4.57]
Ankle	1D	Gravity removal	−2.27[−4.35,−0.22]	−1.06[−5.27,4.54]	1.60[−3.59,7.67]	−0.58[−3.82,3.57]
Ankle	1D	Unprocessed	−2.87[−5.32,−0.48]	−1.53[−5.71,3.91]	3.38[−1.81,9.27]	−0.34[−3.77,3.67]
Hip	Raster	Gravity removal	−1.52[−3.14,0.20]	−3.06[−7.79,2.13]	5.46[−0.54,11.93]	0.29[−3.27,4.25]
Hip	Raster	Unprocessed	−1.98[−3.31,−0.62]	−4.51[−8.92,0.47]	2.03[−5.62,9.57]	−1.49[−5.13,2.55]
Hip	1D	Gravity removal	−0.36[−2.57,1.85]	−1.73[−5.98,3.25]	3.05[−3.31,9.96]	0.32[−3.28,4.39]
Hip	1D	Unprocessed	−3.07[−5.16,−1.12]	−3.59[−11.67,3.30]	7.62[−4.33,19.36]	0.32[−6.20,5.97]
Wrist	Raster	Gravity removal	−3.02[−5.88,−0.64]	−5.13[−9.44,−0.60]	2.29[−6.43,12.61]	−1.95[−6.28,2.95]
Wrist	Raster	Unprocessed	−6.31[−13.00,−1.55]	−6.94[−15.06,0.17]	1.75[−9.28,13.35]	−3.83[−11.50,3.24]
Wrist	1D	Gravity removal	−4.88[−7.48,−2.52]	−4.89[−8.99,−0.38]	−0.77[−8.31,7.34]	−3.51[−7.63,0.74]
Wrist	1D	Unprocessed	−6.53[−13.05,−1.24]	−5.03[−13.72,2.97]	−1.51[−10.11,7.74]	−4.36[−11.30,1.88]

**Table 14:** Sensor-location, input-configuration, and training-label comparison on OxWalk. Values are percentages with 95% bootstrap confidence intervals in brackets. Within each sensor location, the lowest MAPE and the MPE closest to zero are shown in bold.

Position	Input	Preprocessing	Training Labels	MAPE	MPE
Hip	1D	Gravity removal	Original	10.24[7.18,13.49]	−1.01[−5.43,3.51]
Hip	1D	Unprocessed	Original	11.53[8.34,15.10]	−4.76[−9.73,−0.18]
Hip	Raster	Gravity removal	Realigned	6.73[4.80,8.91]	0.45[−2.45,3.47]
Hip	Raster	Unprocessed	Original	16.04[10.44,23.75]	−4.48[−11.78,5.16]
Wrist	1D	Gravity removal	Original	14.55[10.91,18.58]	−2.87[−8.85,2.74]
Wrist	1D	Unprocessed	Original	17.02[12.18,22.21]	−5.79[−12.62,1.51]
Wrist	Raster	Unprocessed	Realigned	13.06[9.79,16.58]	−2.78[−7.95,2.60]
Wrist	Raster	Gravity removal	Realigned	16.25[12.41,20.46]	1.48[−4.71,8.15]
Wrist	Raster	Unprocessed	Original	14.07[10.47,17.85]	−3.10[−8.77,2.50]

**Table 15:** Zero-shot MAPE on Clemson after parameter estimation or training on OxWalk. Values are percentages with 95% bootstrap confidence intervals in brackets. The lowest MAPE in each column is shown in bold.

Method	Regular	Semi-regular	Irregular	Overall
Peak detector	42.48[37.64,47.26]	42.95[38.94,46.45]	32.11[23.76,41.36]	39.18[36.27,42.28]
Threshold detector	57.65[49.67,65.47]	71.87[66.07,77.13]	74.65[68.60,80.31]	68.06[63.08,72.91]
Autocorrelation detector	0.68[0.46,0.92]	13.76[10.06,17.60]	50.46[42.21,58.72]	21.63[18.46,24.87]
Stepcount SSL	4.25[2.83,6.11]	10.68[8.74,12.68]	14.44[9.75,19.82]	9.79[8.03,11.69]
Temporal step supervision	12.97[6.98,20.40]	13.04[9.37,17.05]	16.56[11.34,23.46]	14.19[10.63,18.40]

**Table 16:** Zero-shot MPE on Clemson after parameter estimation or training on OxWalk. Values are percentages with 95% bootstrap confidence intervals in brackets. The MPE closest to zero in each column is shown in bold.

Method	Regular	Semi-Regular	Irregular	Overall
Peak detector	−42.48[−47.26,−37.58]	−42.95[−46.48,−39.01]	30.46[21.13,40.21]	−18.33[−22.86,−13.78]
Threshold detector	−57.65[−65.53,−49.55]	−71.87[−77.20,−66.06]	−74.65[−80.42,−68.73]	−68.06[−72.92,−63.08]
Autocorrelation detector	0.23[−0.10,0.55]	−7.26[−12.88,−1.59]	−27.72[−44.16,−9.31]	−11.59[−18.32,−4.45]
Stepcount SSL	−3.83[−5.83,−2.25]	−9.53[−12.15,−6.80]	3.87[−3.23,11.09]	−3.16[−6.29,0.01]
Temporal step supervision	−7.84[−16.35,−0.95]	−8.68[−14.10,−3.52]	−1.11[−8.76,8.41]	−5.88[−10.95,−0.76]

**Table 17:** Zero-shot transfer from Clemson to OxWalk. Values are percentages with 95% bootstrap confidence intervals in brackets. The lowest MAPE and the MPE closest to zero are shown in bold.

Method	MAPE	MPE
Peak detector	122.22[77.36,176.61]	122.10[77.90,177.64]
Threshold detector	791.55[515.60,1124.18]	790.67[515.60,1116.14]
Autocorrelation detector	17.28[12.64,22.38]	−7.31[−14.17,−0.17]
Stepcount SSL	24.85[12.61,42.37]	21.12[7.99,38.86]
Temporal step supervision	28.75[15.08,48.08]	10.89[−5.24,32.14]

**Table 18:** Matched in-domain and zero-shot MAPE on OxWalk at 15 Hz. Values are percentages with 95% participant-level bootstrap confidence intervals in brackets. The increase is reported in percentage points (pp) and is computed from paired participant-level APE differences.

Method	Matched in-Domain MAPE	Zero-Shot MAPE	Increase
Temporal step supervision	17.38[11.68,24.42]	28.75[15.08,48.08]	+11.37[1.19,24.84] pp
Stepcount SSL	13.96[10.44,17.82]	24.85[12.61,42.37]	+10.89[−0.89,26.67] pp

**Table 19:** Model size and CPU inference time for a 60 s recording. Inference time is reported as mean ± SD.

Method	Parameters	CPU Time (ms)
Temporal step supervision	1,045,177	86.91±2.15
CNN	109,698	12.95±0.68
WaveNet	16,002	46.03±2.01
LSTM	208,578	203.54±3.07
Fine-tuned Stepcount SSL	10,983,234	48.23±0.94

## Data Availability

The Clemson Pedometer dataset analyzed in this study is available at https://cecas.clemson.edu/~ahoover/pedometer/ (accessed on 12 August 2026). The OxWalk dataset analyzed in this study is available through the Oxford Research Archive at https://ora.ox.ac.uk/objects/uuid:19d3cb34-e2b3-4177-91b6-1bad0e0163e7 (accessed on 12 August 2026). The complete implementation and experiment configurations are publicly available at https://github.com/yanzhang0727/temporal-step-supervision (accessed on 12 August 2026).

## Abbreviations

The following abbreviations are used in this manuscript:

APE	Absolute percentage error
BCE	Binary cross entropy
CNN	Convolutional neural network
CV	Coefficient of variation
ISI	Inter-step interval
LSTM	Long short-term memory
MAPE	Mean absolute percentage error
MPE	Mean percentage error
RF	Random forest
SSL	Self-supervised learning
U-Net	U-shaped network

## Appendix A. Implementation Details

### Appendix A.1. Dense Label Width

The dense label width was w=5 for Clemson at 15 Hz and w=7 for OxWalk at 25 Hz, approximately one third of a second in both datasets. This width was specified to cover the local acceleration response around each annotated step while limiting overlap between adjacent step labels.

### Appendix A.2. Rasterization Details

For each dataset, sensor location, fold, and acceleration axis *c*, the lower and upper raster limits $v_{\min,c}$ and $v_{\max,c}$ were set to the 0.5th and 99.5th percentiles of the preprocessed acceleration values among the training participants within that fold. Validation and test samples were clipped to these training-derived limits and quantized into H=16 uniformly spaced amplitude bins. Percentile-based limits were used to reduce the influence of extreme acceleration values. The raster quantization used $\epsilon =10^{-8}$ to prevent division by zero.

For non-rasterized 1D runs, the same training-fold limits were used to clip and scale the tri-axial window to approximately [−1,1].

### Appendix A.3. Network Hyperparameters

The raster U-Net channel widths were selected according to the input temporal resolution induced by the dataset sampling rate. For Clemson, sampled at 15 Hz, the encoder channel widths were 24, 48, and 96, and the bottleneck used 192 channels. For OxWalk, sampled at 25 Hz, the encoder channel widths were 32, 64, and 128, and the bottleneck used 256 channels. The larger channel configuration for OxWalk reflects its longer 60-s input windows at the higher sampling rate.

Each encoder block contains two 3×3 convolutional layers with batch normalization and GELU activation. Spatial dropout in the two-dimensional convolutional blocks used p=0.1. Downsampling was applied only along the amplitude-bin dimension using max pooling with kernel size (2,1), so the temporal resolution was preserved throughout the encoder. The decoder restored the amplitude resolution using transposed convolutions with kernel size (2,1) and skip connections.

After decoding, the amplitude dimension was collapsed using a convolution with kernel size (3,1). A temporal refinement layer with kernel size (1,9) was then applied before the final sigmoid output. The raster model therefore maps an input tensor of shape (B,3,H,T) to a temporal probability sequence of shape (B,T).

### Appendix A.4. Training Hyperparameters

Table A1 reports the training settings used for the reported models.

**Table A1:** Training hyperparameters.

Hyperparameter	Value
Optimizer	Adam
Learning rate	$3\times 10^{-4}$
Weight decay	$1\times 10^{-4}$
Batch size	64
Maximum epochs	120
Early-stopping patience	20 epochs
Learning rate schedule	Cosine annealing
Minimum learning rate	$1\times 10^{-5}$
Gradient clipping	Norm 1.0
Count loss maximum weight $\alpha_{\max}$	0.15
Count loss warmup $E_{\mathrm{warmup}}$	30 epochs
Count loss ramp $E_{\mathrm{ramp}}$	50 epochs

### Appendix A.5. Input Preprocessing

The model input was tri-axial acceleration from the selected sensor location. In experiments that used gravity removal, each accelerometer axis was processed independently. A fourth-order Butterworth low-pass filter with cutoff 1 Hz was applied using zero-phase forward-backward filtering to estimate the gravity and posture component [39]. This component was subtracted from the raw signal:

$${\tilde{x}}_{c}\left(t\right)=x_{c}\left(t\right)-{\mathrm{LPF}}_{1\mathrm{Hz}}\left\{x_{c}\right\}\left(t\right),$$

where *c* indexes the acceleration axis.

The selected tri-axial signal, with or without gravity removal, was then passed to the raster or 1D representation step.

### Appendix A.6. Motion-Energy Label Realignment

Motion-energy label realignment was used because dense temporal supervision is sensitive to timing mismatch between annotated step times and accelerometer measurements. A manual step timestamp marks a gait event, but the nearby accelerometer response may peak slightly before or after the annotation time.

To align the training target with local accelerometer evidence, we applied a local realignment procedure to the training annotations. For realignment only, each acceleration axis was filtered with a fourth-order Butterworth band-pass filter from 0.3 to 4.0 Hz using zero-phase forward-backward filtering [39]. A motion energy trace was computed as

$$E\left(t\right)=\sum_{c=1}^{3}x_{c,\mathrm{bp}}{\left(t\right)}^{2}.$$

For each annotated step at sample $t_{j}$, the algorithm searched within ±0.25 s of $t_{j}$ and moved the label to the sample with maximum motion energy in that local window. If multiple annotations mapped to the same sample, the nearest unused sample inside the same search window was selected to preserve the number of annotations.

Realignment was applied only to training annotations used for dense temporal label construction. Validation and test annotations, reference counts, and accelerometer signals were left unchanged.

## Appendix B. CPU Inference Benchmark

All inference times were measured on CPU using one intra-op and one inter-op thread, with batch size 1, after 20 warm-up runs and over 100 timed repeats. Because the evaluated models use different native input formats, timing was defined as the complete inference pipeline required to process an equivalent 60-s recording.

For temporal step supervision, the benchmark used one 60-s raster input of shape 1×3×16×900.

For CNN and WaveNet, inference was performed using sliding 50-sample windows over the 60-s 15-Hz recording. For LSTM, sliding 30-sample windows were used. The reported times for CNN, WaveNet, and LSTM include sliding-window classification, prediction smoothing, and conversion of the predicted state sequence to a step count.

For fine-tuned Stepcount SSL, the 60-s recording was processed as six 10-s windows resampled to 30 Hz. The reported time includes walking-window classification, low-pass filtering of the acceleration vector magnitude, and peak-counting post-processing.

## Appendix C. Reproduction of the Prior Clemson Evaluation Protocol

To verify the implementation of the CNN, WaveNet, and LSTM reference pipelines, we reproduced the evaluation protocol used in prior Clemson step-counting work [23]. Clemson wrist recordings were evaluated using 15 leave-two-participants-out folds:

(P001,P002),(P003,P004),…,(P029,P030).

The reproduced pipelines predict left/right step states from short sliding windows and obtain step counts from transitions in the predicted state sequence. The reproduced protocol included checkpoint selection using the participants subsequently evaluated in each fold, following the procedure of the original study. This appendix is included only to verify the reimplementation of the CNN, WaveNet, and LSTM pipelines; no main conclusion of this study relies on this evaluation protocol.

Table A2 reports the reproduction results together with temporal step supervision evaluated under the same protocol. The reproduced CNN, WaveNet, and LSTM pipelines achieved MAPE values of 1.71%, 1.26%, and 2.97% under regular walking. Their errors increased to 106.83%, 292.65%, and 295.57% under irregular walking. Temporal step supervision obtained 2.33% MAPE under regular walking and 12.05% under irregular walking.

**Table A2:** Reproduction of CNN, WaveNet, and LSTM reference pipelines on Clemson wrist recordings using the prior leave-two-participants-out evaluation protocol. Values are MAPE (%), mean ± SD. The best result in each column is shown in bold.

Method	Regular	Semi-Regular	Irregular
Reproduced CNN pipeline	1.71±3.42	16.59±16.10	106.83±75.81
Reproduced WaveNet pipeline	1.26±1.03	31.00±17.01	292.65±122.44
Reproduced LSTM pipeline	2.97±3.71	25.60±16.70	295.57±131.32
Temporal step supervision	2.33±2.12	8.61±10.05	12.05±20.70

## Appendix D. Annotation-Derived Timing Complexity Analysis

To characterize the temporal organization of the annotated windows, we computed summary statistics from step annotations. For Clemson, statistics were computed for the original shared annotations and for the sensor-specific peakenergy025 annotations for wrist and hip. For OxWalk, statistics were computed from the original wrist annotations. These statistics were used only for analysis and were not used for training, checkpoint selection, or test evaluation.

Inter-step intervals were defined as

$${\mathrm{ISI}}_{k}=t_{k+1}-t_{k},$$

where $t_{k}$ and $t_{k+1}$ are consecutive annotated step times within a 60-s window. Inter-step interval variability was computed only for windows with at least five annotated steps:

$${\mathrm{CV}}_{\mathrm{ISI}}=\frac{\mathrm{std}(\mathrm{ISI})}{\mathrm{mean}(\mathrm{ISI})}.$$

Sparse windows were defined as windows with 0<C<10, where *C* is the reference step count. Long gaps were defined as inter-step intervals greater than 2 s. Walking bouts were defined by splitting consecutive annotated steps whenever the inter-step interval exceeded 2 s.

Table A3 summarizes the overall timing statistics. Table A4 compares timing statistics within matched reference-count bins.

**Table A3:** Annotation-derived timing statistics for 60-s windows. Sparse windows were defined as 0<C<10. ISI CV was computed only for windows with C≥5. Long gaps were defined as inter-step intervals greater than 2 s.

Dataset	Annotation	Condition	*n* Windows	Zero Frac.	Sparse Frac.	Median Count	ISI CV Mean	Long-Gap Frac.	Bout Count	Median Bout Length
Clemson	Original shared	Regular	312	0.016	0.000	110	0.088	0.000	0.990	110.00
Clemson	Original shared	Semi-regular	303	0.017	0.007	68	0.944	0.054	3.799	14.00
Clemson	Original shared	Irregular	486	0.222	0.144	12	0.752	0.025	1.035	9.50
Clemson	Wrist peakenergy025	Regular	312	0.016	0.000	110	0.486	0.000	0.994	110.00
Clemson	Wrist peakenergy025	Semi-regular	303	0.017	0.007	68	1.031	0.054	3.805	14.00
Clemson	Wrist peakenergy025	Irregular	486	0.224	0.136	12	0.903	0.027	1.045	9.00
Clemson	Hip peakenergy025	Regular	312	0.016	0.000	110	0.518	0.000	0.990	110.00
Clemson	Hip peakenergy025	Semi-regular	303	0.017	0.007	68	1.025	0.055	3.875	13.00
Clemson	Hip peakenergy025	Irregular	486	0.222	0.138	12	0.902	0.029	1.066	8.75
OxWalk	Original wrist	Free-living	2276	0.528	0.072	0	0.883	0.077	1.166	0.00

The Clemson original annotations are shared across sensor locations. The peakenergy025 annotations are sensor-specific because each annotation is moved to a nearby local energy peak computed from the selected sensor signal. Adding the Clemson wrist and hip peakenergy025 annotations did not change the main pattern: OxWalk had many more zero-step windows, and within low and moderate count bins OxWalk showed larger long-gap fractions and shorter walking bouts than Clemson.

**Table A4:** Timing statistics within matched reference-count bins. Long gaps were defined as inter-step intervals greater than 2 s, and walking bouts were split at long gaps.

Count Bin	Annotation	Condition	ISI CV	Long-Gap Frac.	Bout Count	Median Bout Length
1–10	Clemson original shared	Irregular	0.563	0.037	1.238	5.00
1–10	Clemson wrist peakenergy025	Irregular	0.797	0.044	1.277	5.00
1–10	Clemson hip peakenergy025	Irregular	0.771	0.040	1.238	5.00
1–10	OxWalk original wrist	Free-living	1.115	0.280	1.972	2.00
11–30	Clemson original shared	Irregular	0.738	0.022	1.331	14.00
11–30	Clemson wrist peakenergy025	Irregular	0.875	0.023	1.345	14.00
11–30	Clemson hip peakenergy025	Irregular	0.879	0.026	1.380	13.00
11–30	OxWalk original wrist	Free-living	1.471	0.151	3.714	4.50
31–60	Clemson original shared	Semi-regular	1.157	0.098	5.427	7.00
31–60	Clemson wrist peakenergy025	Semi-regular	1.187	0.101	5.568	6.00
31–60	Clemson hip peakenergy025	Semi-regular	1.192	0.101	5.551	7.00
31–60	OxWalk original wrist	Free-living	1.467	0.073	3.859	9.00

Overall, OxWalk had substantially more zero-step windows than Clemson. More than half of OxWalk windows contained no annotated steps, whereas zero-step windows were rare in the Clemson regular and semi-regular conditions. The median OxWalk window count was also zero, indicating that many free-living windows contained no walking or only sparse walking.

Within matched count bins, OxWalk windows had higher long-gap fractions and shorter walking bouts than Clemson irregular windows in the 1–10 and 11–30 step ranges. This indicates that similar counts can correspond to more fragmented temporal step arrangements in OxWalk. These annotation-derived statistics support the interpretation that aggregate count supervision is less reliable when walking is sparse or intermittent.

## Appendix E. Complete Preprocessing and Label-Construction Results

This appendix reports the complete preprocessing and label-construction results under the unified evaluation protocol. Table A5 and Table A6 report the complete Clemson results, and Table A7 reports the complete OxWalk results. The tables include both original labels and motion-energy realigned labels. Clemson results use the same ten participant splits as the main experiments, and OxWalk results use the same subject-wise 10-fold evaluation. Values are reported as MAPE and MPE with 95% bootstrap confidence intervals.

Under the unified protocol, the effect of motion-energy realignment depended on the sensor location and preprocessing configuration. For Clemson raster input with unprocessed acceleration, overall MAPE changed from 15.89% to 16.18% at the wrist, from 10.31% to 8.86% at the hip, and from 8.40% to 8.22% at the ankle. After gravity removal, the corresponding changes were 10.10% to 11.13%, 9.49% to 9.03%, and 8.08% to 8.11%, respectively.

On OxWalk, motion-energy realignment reduced raster-input MAPE from 14.07% to 13.06% at the wrist and from 16.04% to 10.57% at the hip with unprocessed acceleration. After gravity removal, MAPE decreased from 19.04% to 16.25% at the wrist and from 8.94% to 6.73% at the hip. These results indicate that the effect of label realignment depends on the dataset, sensor location, input representation, and acceleration preprocessing.

**Table A5:** Complete Clemson MAPE (%) results under the unified evaluation protocol. Values in brackets are 95% participant-level bootstrap confidence intervals. Overall intervals use participant-cluster resampling, retaining all three gait conditions for each sampled participant. Within each sensor location, the lowest MAPE in each column is shown in bold.

Position	Input	Labels	Input Preprocessing	Regular	Semi-Regular	Irregular	Overall
Wrist	1D	original	unprocessed	12.53[5.91,20.98]	18.95[12.17,27.28]	23.37[17.56,29.53]	18.28[12.68,25.04]
Wrist	1D	original	gravity removal	6.88[5.07,8.86]	12.14[8.74,15.98]	16.81[12.63,21.93]	11.94[9.50,14.73]
Wrist	1D	motion-energy realigned	unprocessed	10.25[5.82,16.08]	17.67[12.31,23.69]	19.55[14.37,24.93]	15.82[11.93,20.53]
Wrist	1D	motion-energy realigned	gravity removal	6.20[4.28,8.31]	10.50[7.81,13.40]	16.59[12.02,21.94]	11.10[8.59,13.93]
Wrist	Raster	original	unprocessed	9.46[4.65,16.54]	15.00[9.50,22.14]	23.22[16.25,31.02]	15.89[11.18,21.99]
Wrist	Raster	original	gravity removal	4.61[2.98,6.99]	9.90[6.80,13.22]	15.78[10.11,23.20]	10.10[7.30,13.66]
Wrist	Raster	motion-energy realigned	unprocessed	8.94[4.62,15.24]	15.69[10.65,22.15]	23.90[16.85,31.19]	16.18[11.74,21.71]
Wrist	Raster	motion-energy realigned	gravity removal	5.57[3.84,7.82]	9.90[6.88,13.30]	17.92[11.96,26.06]	11.13[8.29,14.73]
Hip	1D	original	unprocessed	5.23[3.47,7.37]	12.16[7.13,19.18]	23.09[12.43,36.78]	13.50[8.28,19.90]
Hip	1D	original	gravity removal	3.72[2.62,5.02]	9.17[5.81,13.20]	14.61[10.80,19.11]	9.17[6.75,12.11]
Hip	1D	motion-energy realigned	unprocessed	4.69[3.23,6.36]	10.59[5.34,17.86]	23.20[14.95,32.80]	12.83[8.51,18.17]
Hip	1D	motion-energy realigned	gravity removal	4.52[3.12,6.10]	8.56[5.32,12.50]	14.39[10.48,19.23]	9.16[6.84,11.98]
Hip	Raster	original	unprocessed	4.86[3.47,6.47]	11.15[8.08,14.70]	14.92[9.51,21.71]	10.31[8.01,13.21]
Hip	Raster	original	gravity removal	3.53[2.39,4.81]	9.96[6.54,14.17]	14.99[10.68,20.43]	9.49[7.22,12.47]
Hip	Raster	motion-energy realigned	unprocessed	3.13[2.16,4.16]	9.75[6.55,13.69]	13.70[8.60,20.03]	8.86[6.50,11.85]
Hip	Raster	motion-energy realigned	gravity removal	3.83[2.80,5.01]	9.78[6.45,13.75]	13.49[9.56,18.31]	9.03[6.80,11.81]
Ankle	1D	original	unprocessed	5.07[3.81,6.41]	8.92[5.34,13.75]	12.32[8.64,16.83]	8.77[6.58,11.59]
Ankle	1D	original	gravity removal	3.06[2.11,4.10]	8.02[4.17,13.18]	11.90[7.97,16.88]	7.66[5.19,10.97]
Ankle	1D	motion-energy realigned	unprocessed	5.62[4.04,7.36]	9.14[6.06,13.24]	11.11[7.62,15.66]	8.62[6.45,11.47]
Ankle	1D	motion-energy realigned	gravity removal	4.56[3.23,6.12]	8.56[5.22,12.96]	10.59[6.93,15.25]	7.90[5.61,10.96]
Ankle	Raster	original	unprocessed	4.68[3.46,6.00]	8.87[5.56,13.26]	11.66[8.02,16.21]	8.40[6.14,11.31]
Ankle	Raster	original	gravity removal	3.04[2.03,4.27]	9.02[5.40,13.64]	12.17[8.40,17.05]	8.08[5.76,11.17]
Ankle	Raster	motion-energy realigned	unprocessed	3.92[2.67,5.39]	8.40[5.02,12.89]	12.34[8.32,17.06]	8.22[5.86,11.25]
Ankle	Raster	motion-energy realigned	gravity removal	3.70[2.64,4.94]	8.34[4.63,12.80]	12.29[8.45,17.12]	8.11[5.66,11.21]

**Table A6:** Complete Clemson MPE (%) results under the unified evaluation protocol. Values in brackets are 95% participant-level bootstrap confidence intervals. Overall intervals use participant-cluster resampling, retaining all three gait conditions for each sampled participant. Within each sensor location, the MPE closest to zero in each column is shown in bold.

Position	Input	Labels	Input Preprocessing	Regular	Semi-Regular	Irregular	Overall
Wrist	1D	original	unprocessed	−8.76[−17.74,−1.49]	−9.27[−18.95,−0.30]	−2.92[−13.10,7.63]	−6.98[−15.95,0.91]
Wrist	1D	original	gravity removal	−1.02[−4.20,1.95]	−4.42[−9.85,1.00]	−0.02[−7.15,8.03]	−1.82[−6.22,2.76]
Wrist	1D	motion-energy realigned	unprocessed	−6.53[−13.05,−1.24]	−5.03[−13.72,2.97]	−1.51[−10.11,7.74]	−4.36[−11.30,1.88]
Wrist	1D	motion-energy realigned	gravity removal	−4.88[−7.48,−2.52]	−4.89[−8.99,−0.38]	−0.77[−8.31,7.34]	−3.51[−7.63,0.74]
Wrist	Raster	original	unprocessed	−8.28[−15.55,−3.16]	−8.80[−16.89,−1.65]	0.88[−9.92,12.30]	−5.40[−13.25,1.58]
Wrist	Raster	original	gravity removal	−2.33[−5.10,−0.13]	−5.28[−9.55,−0.62]	2.87[−5.07,12.44]	−1.58[−5.69,3.12]
Wrist	Raster	motion-energy realigned	unprocessed	−6.31[−13.00,−1.55]	−6.94[−15.06,0.17]	1.75[−9.28,13.35]	−3.83[−11.50,3.24]
Wrist	Raster	motion-energy realigned	gravity removal	−3.02[−5.88,−0.64]	−5.13[−9.44,−0.60]	2.29[−6.43,12.61]	−1.95[−6.28,2.95]
Hip	1D	original	unprocessed	−1.24[−4.14,1.23]	−6.71[−14.06,−0.29]	5.39[−8.22,21.16]	−0.86[−7.36,5.39]
Hip	1D	original	gravity removal	−0.38[−2.12,1.49]	−2.55[−7.00,2.80]	0.55[−6.02,7.48]	−0.79[−4.49,3.20]
Hip	1D	motion-energy realigned	unprocessed	−3.07[−5.16,−1.12]	−3.59[−11.67,3.30]	7.62[−4.33,19.36]	0.32[−6.20,5.97]
Hip	1D	motion-energy realigned	gravity removal	−0.36[−2.57,1.85]	−1.73[−5.98,3.25]	3.05[−3.31,9.96]	0.32[−3.28,4.39]
Hip	Raster	original	unprocessed	−4.66[−6.35,−3.18]	−6.43[−10.77,−1.32]	1.19[−7.30,9.04]	−3.30[−7.10,0.70]
Hip	Raster	original	gravity removal	−1.88[−3.48,−0.17]	−2.73[−7.45,2.89]	4.93[−1.78,12.38]	0.11[−3.76,4.57]
Hip	Raster	motion-energy realigned	unprocessed	−1.98[−3.31,−0.62]	−4.51[−8.92,0.47]	2.03[−5.62,9.57]	−1.49[−5.13,2.55]
Hip	Raster	motion-energy realigned	gravity removal	−1.52[−3.14,0.20]	−3.06[−7.79,2.13]	5.46[−0.54,11.93]	0.29[−3.27,4.25]
Ankle	1D	original	unprocessed	1.12[−1.05,3.34]	−0.78[−5.27,5.04]	2.42[−3.19,8.72]	0.92[−2.38,4.85]
Ankle	1D	original	gravity removal	0.35[−1.17,1.81]	−0.02[−4.70,6.09]	2.05[−3.59,8.36]	0.80[−2.52,5.03]
Ankle	1D	motion-energy realigned	unprocessed	−2.87[−5.32,−0.48]	−1.53[−5.71,3.91]	3.38[−1.81,9.27]	−0.34[−3.77,3.67]
Ankle	1D	motion-energy realigned	gravity removal	−2.27[−4.35,−0.22]	−1.06[−5.27,4.54]	1.60[−3.59,7.67]	−0.58[−3.82,3.57]
Ankle	Raster	original	unprocessed	−3.38[−5.08,−1.68]	−2.06[−6.22,3.48]	3.53[−1.94,9.64]	−0.64[−3.94,3.35]
Ankle	Raster	original	gravity removal	−1.68[−3.22,−0.31]	−1.81[−6.33,4.02]	2.54[−3.07,9.11]	−0.32[−3.76,3.94]
Ankle	Raster	motion-energy realigned	unprocessed	−2.87[−4.62,−1.26]	−1.62[−5.83,3.81]	6.16[0.46,12.41]	0.55[−2.85,4.57]
Ankle	Raster	motion-energy realigned	gravity removal	−2.54[−4.12,−1.09]	−1.56[−6.17,3.88]	4.29[−1.37,10.66]	0.06[−3.48,4.16]

**Table A7:** Complete OxWalk results for input representation, label construction, and acceleration preprocessing under the unified subject-wise 10-fold evaluation. Values are MAPE and MPE (%) with 95% bootstrap confidence intervals. Within each sensor location, the lowest MAPE and the MPE closest to zero are shown in bold.

Position	Input	Labels	Input Preprocessing	MAPE (%) [95% CI]	MPE (%) [95% CI]
Wrist	1D	original	unprocessed	17.02[12.18,22.21]	−5.79[−12.62,1.51]
Wrist	1D	original	gravity removal	14.55[10.91,18.58]	−2.87[−8.85,2.74]
Wrist	1D	motion-energy realigned	unprocessed	16.10[12.26,20.31]	−4.66[−11.11,1.53]
Wrist	1D	motion-energy realigned	gravity removal	14.50[10.87,18.33]	−1.40[−7.36,4.55]
Wrist	Raster	original	unprocessed	14.07[10.47,17.85]	−3.10[−8.77,2.50]
Wrist	Raster	original	gravity removal	19.04[14.43,24.34]	1.66[−6.00,9.73]
Wrist	Raster	motion-energy realigned	unprocessed	13.06[9.79,16.58]	−2.78[−7.95,2.60]
Wrist	Raster	motion-energy realigned	gravity removal	16.25[12.41,20.46]	1.48[−4.71,8.15]
Hip	1D	original	unprocessed	11.53[8.34,15.10]	−4.76[−9.73,−0.18]
Hip	1D	original	gravity removal	10.24[7.18,13.49]	−1.01[−5.43,3.51]
Hip	1D	motion-energy realigned	unprocessed	12.00[8.25,16.21]	−4.26[−9.51,1.03]
Hip	1D	motion-energy realigned	gravity removal	9.01[6.48,11.95]	1.10[−2.87,5.06]
Hip	Raster	original	unprocessed	16.04[10.44,23.75]	−4.48[−11.78,5.16]
Hip	Raster	original	gravity removal	8.94[6.59,11.60]	−0.69[−4.44,3.16]
Hip	Raster	motion-energy realigned	unprocessed	10.57[7.50,14.23]	−1.33[−5.86,3.55]
Hip	Raster	motion-energy realigned	gravity removal	6.73[4.80,8.91]	0.45[−2.45,3.47]
